# Urotensin-II Ligands: An Overview from Peptide to Nonpeptide Structures

**DOI:** 10.1155/2013/979016

**Published:** 2013-02-25

**Authors:** Francesco Merlino, Salvatore Di Maro, Ali Munaim Yousif, Michele Caraglia, Paolo Grieco

**Affiliations:** ^1^Department of Pharmacy, University of Naples Federico II, 80131 Naples, Italy; ^2^Department of Biochemistry, Biophysics and General Pathology, Second University of Naples, 80138 Naples, Italy

## Abstract

Urotensin-II was originally isolated from the goby urophysis in the 1960s as a vasoactive peptide with a prominent role in cardiovascular homeostasis. The identification of human isoform of urotensin-II and its specific UT receptor by Ames et al. in 1999 led to investigating the putative role of the interaction U-II/UT receptor in multiple pathophysiological effects in humans. Since urotensin-II is widely expressed in several peripheral tissues including cardiovascular system, the design and development of novel urotensin-II analogues can improve knowledge about structure-activity relationships (SAR). In particular, since the modulation of the U-II system offers a great potential for therapeutic strategies related to the treatment of several diseases, like cardiovascular diseases, the research of selective and potent ligands at UT receptor is more fascinating. In this paper, we review the developments of peptide and nonpeptide U-II structures so far developed in order to contribute also to a more rational and detectable design and synthesis of new molecules with high affinity at the UT receptor.

## 1. Introduction

**Figure 1:**
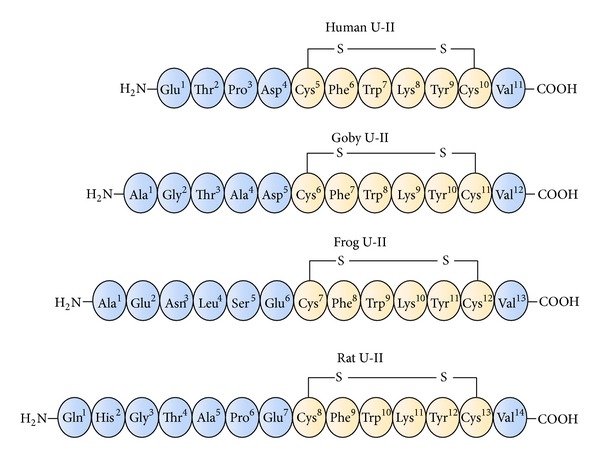
A comparison of urotensin-II (U-II) isopeptides sequences isolated from different species of vertebrates.

Urotensin-II (U-II) belongs to a series of regulatory neuropeptides first isolated from the urophysis of the teleost fish *Gillichthys mirabilis* by Karl Lederis and Howard Bern in the 1960s. This cyclic peptide derived from goby fish, H-Ala-Gly-Thr-Ala-Asp-c[Cys-Phe-Trp-Lys-Tyr-Cys]-Val-OH, was originally characterized on the basis of its interesting smooth muscle contracting and hypertensive effects. It has been long considered that U-II was exclusively produced by the fish urophysis [1]. However, the identification of U-II from the brain of a frog [2, 3] has shown that the cDNA encoding prepro-U-II exists in several species of vertebrates. Moreover, the gene is expressed not only in the caudal portion of the spinal cord but also in brain neurones, from frogs to humans [4]. In fact, urotensin-II isopeptides are present in several species of vertebrates, and although the amino acid sequence in the N-terminus of urotensin-II peptides diverges across species, the cyclic hexapeptide sequence, c[Cys-Phe-Trp-Lys-Tyr-Cys], is conserved in all isoforms (Figure 1, orange residues).

The length of urotensin-II peptides is variable across species and it ranged from 17 amino acid residues in mice to 11 in humans, depending on the proteolytic cleavages that occur in precursors. The N-terminus region of U-II is highly variable among animal species [5], whereas the C-terminal amino acids, organized in a disulphide-linked cyclic array, c[Cys-Phe-Trp-Lys-Tyr-Cys], are continuously conserved from species to species, suggesting their primary role in the biological activity [6]. In addition, the goby isoform of U-II exhibits some structural similarities with somatostatin-14 concerning especially the presence of a disulphide-linked cyclic core at their C-terminus portion containing the biologically active domain Phe-Trp-Lys [7]. Moreover, the characterization of cDNA encoding carp pro-U-II has shown that the U-II and somatostatin-14 precursors share a common structure organization since the active peptides are both located at the C-terminal portion of the precursors [8]. Later, once the UT receptor was identified as a member of somatostatin receptor family, some somatostatin-like peptides containing a disulphide bridge, such as human melanin-concentrating hormone (MCH), somatostatin-14, cortistatin-14, and octreotide, were screened on UT receptor in order to compare the resulting biological activities with that of the endogenous U-II [9]. By these observations it was established that U-II represented the only endogenous ligand with high affinity for the somatostatin-like receptor named UT receptor.

The human UT-II (hU-II) is a cyclic undecapeptide, H-Glu-Thr-Pro-Asp-c[Cys-Phe-Trp-Lys-Tyr-Cys]-Val-OH, recognized as the natural ligand of an orphan G-protein coupled receptor, first named as a rat receptor with high affinity for U-II, GPR14 [10–12]. Subsequently, a human G-protein coupled receptor showing 75% similarity to the orphan rat receptor was cloned and renamed UT receptor by IUPHAR [13]. The role of UT receptor has been continuously investigated and nowadays it is commonly accepted that it is widely distributed in the CNS and in different peripheral tissues including cardiovascular system [14], kidney, bladder, prostate, and adrenal gland [10, 15–17]. This extensive expression has revealed the multiple pathophysiological effects mediated by the hUT/UT receptor interaction such as cardiovascular disorders (heart failure, cardiac remodelling, and atherosclerosis), smooth muscle cell proliferation, renal disease, diabetes, and tumour growth [18]. Nevertheless, UT receptor is especially expressed in vascular smooth muscle, endothelium, and myocardium and plays a key role in the regulation of the cardiovascular homeostasis. Furthermore, this receptor has much structural homology to members of the somatostatin and receptor family and it could be activated by somatostatin-14 and cortistatin at micromolar doses [10]. The gene coding for the UT receptor has been located in chromosome 17q25.3 [19].

The hU-II binds with high affinity to this receptor, resulting in intracellular calcium mobilization via phospholipase C-dependent increase in inositol phosphates. In isolated rat thoracic aorta fragments hU-II induces contraction mediated by two distinct tonic and phasic components. However, its vasoconstrictor activity is well observed in primate arteries, in which it causes a concentration-dependent contraction of isolated arterial rings with an EC_50_ value less than 1 nM, meaning a 10-fold more potency than endothelin-1. However, the *in vivo* effects can depend on the species, type of blood vessel, concentration of U-II, route of administration, and results of tissue and species in exam, and they can be also contradictory. Accordingly, the peptide also elicits vasodilatory effects on the small arteries of rats and on the resistance arteries of humans, probably due to the release of endothelium-derived hyperpolarizing factor and nitric oxide [20]. In a healthy human, U-II behaves as a chronic regulator of vascular tone rather than influencing tissues in a phasic manner [21]. U-II binds to its receptor in a “pseudo-irreversible” manner, and slow dissociation rate from the UT receptor leads to prolonged activation of the receptor and a functionally silent system [22]. This state of homeostasis is altered since pathogenesis of several cardiovascular disorders provokes an upregulation of UT receptor and of U-II resulting in vasoconstriction.

To date, findings in molecules with high affinity to the urotensin-II system has led to discovering new ligands, peptide, and nonpeptide analogues. This review is aimed at investigating the structure-activity relationship on urotensin-II system by analyzing peptide and nonpeptide structures so far developed in order to contribute also to a more rational and detectable design and synthesis of new molecules with high affinity at the UT receptor.

## 2. SAR Studies on U-II

The potential therapeutic application of urotensin-II system has continuously stimulated structure-activity relationship studies (SARs), which could elucidate the structural features of this important hormone. On the other hand, the discovery of new analogues acting as agonist or antagonist is extremely important to explore the physiological role of hU-II. First structural studies performed on U-II by nuclear magnetic resonance (NMR) spectroscopy [23–25] have revealed that the peptide adopted a preferential conformation both in DMSO and water solution, which did not show any classical secondary structures. Amino acids in the core region of U-II are identified in a highly compact conformation with the formation of a hydrophobic pocket, in which Ala^4^, Phe^7^, Trp^8^, and Val^12^ are projected on the same side of the molecule being at least in part important residues for the binding site. In contrast, hU-II and some analogues resulted to be fold in defined secondary structure when dissolved in SDS solution, a membrane mimetic environment [26].

Subsequently, with the aim of investigating the role played by the exocyclic region in the receptor interaction, a series of truncated peptides related to hU-II has been investigated. Truncation studies are of prime importance for the detection of the minimal sequence of peptide required to retain the biological activity. The effect of sequential deletion of exocyclic residues from N- or C-termini in hU-II sequence does not appear to be significant in the calcium-mobilizing potency and efficacy, whereas the removal of any residue that belongs to the cyclic region determines reduction or total absence of the biological activity. The shortest and fully potent sequence of U-II was individuated in the octapeptide U-II_(4–11)_, H-Asp-c[Cys-Phe-Trp-Lys-Tyr-Cys]Val-OH, that preserves the potency at the human UT receptor, showing similarity to somatostatin-14 in which truncation of the segment led to active analogues.

The importance of a free amino group in the N-terminal of the resulted octapeptide U-II_(4–11)_ was evaluated by its modification in the succinoyl derivative performed in 2002 by Coy et al. [27]. The peptide was extremely potent according to data from the biological activity, EC_50_ = 0.12 ± 0.03 nM, and binding affinity, *K*_*i*_ = 1.14 ± 0.01 nM and *K*_*i*_ = 1.65 ± 0.04 nM, at human and rat UT receptor, respectively. Additional dicarboxylic residues introduced in substitution of Asp^4^ have showed similar results. Furthermore, the replacement of this amino acid with the corresponding amidated Asn gives a compound that retains a full activity in all three assay systems, suggesting that in this position an amino acid negatively charged in the side chain is not required. However, the Nle^4^-analogue lacks of potency showing that the –CH_2_COX carbonyl group present in both the Asp and Asn side chains is important probably because of its possibility to act as an acceptor for hydrogen bond with the UT receptor. The side chain can also contain an aromatic ring substituted with polar groups such as OH and NO_2_, which is of great interest in the development of antagonists based on the previously identified somatostatin antagonist octapeptides. 

The cyclic structure is essential for hU-II and McMaster et al. in 1986 [28] reported a lack of biological activity for the corresponding “ring-opened” analogue. In 2002 Grieco et al. considered the replacement of the disulphide bridge by a side-chain-to-side-chain lactam bridge in accordance with observations on several biologically relevant peptides, such as conotoxins, endothelin-1, and somatostatin analogue that gave interesting results by the same modification [29]. Starting from the minimum active fragment U-II_(4–11)_, introduction of the lactam bridge in an appropriate length led to peptides that maintain bioactivity to the detriment of potency, suggesting that the size of the lactam bridge is a crucial parameter. Peptide analogues synthesized in this study were characterized by ring-closing sequence that ranges from 20 to 24 atoms and, interestingly, the smallest peptide sequence, having the same length as the native peptide containing the disulphide bridge, does not show any biological activity. In contrast, peptide analogue characterized by a larger ring, containing Orn and Asp as residues in the 22 atoms lactam bridge, behaved as a full agonist, but was approximately 100-fold less potent than hU-II. Thus, replacement of the Cys-Cys cyclic motif could be well tolerated by an appropriate longer lactam bridge despite the partial loss of activity, probably due to the different orientation of the key amino acid side chains. However, in a later work performed by Foister et al. in 2006 [30] a cyclic “cysteine-free” hexapeptide derivative of U-II, in which Tyr^9^ was replaced with a *β*-naphthylalanine residue, [Ala-Phe-Trp-Lys-(2)Nal-Ala], bounds the human UT receptor with higher affinity (*K*_*i*_ = 2.8 nM) than the corresponding disulphide-bridged truncated hexapeptide U-II_(5–10)_ (*K*_*i*_ = 95 nM). Furthermore, modifications of the Cys^5^–Cys^10^ disulphide bridge, such as the macrocyclic lactam and the penicillamine-derived disulphide moiety, could chemically stabilize and restrict the conformational flexibility of the biologically active cyclic hexapeptide core sequence. In particular, penicillamine residue in replacement of Cys^5^ resulted to be very useful in order to obtain a potent agonist, [Pen^5^]hU-II_(4–11)_, subsequently renamed P5U [29]. As potent agonist with reduced conformational flexibility, Lavecchia et al. performed subsequent NMR study in DMSO on this peptide in 2005 [31]. The positions of Lys^8^ amino group and Tyr^9^ aromatic side chain were in proximity with a distance of 6.2 Å and Trp^7^ indole and Lys^8^ amine were separated by 5.6 Å. Docking of P5U into a hUT homology model based on the structure of bovine rhodopsin revealed interesting points of interaction. The Lys^8^ interacts primarily with Asp^130^, as well as with residues of Tyr^100^ and Tyr^305^; Tyr^9^ is accommodated in a binding pocket defined by Lys^212^, Val^296^, Ala^281^, and Trp^277^ particularly involved in a *π*-stacking interaction, and the indole NH of Trp^7^ binds to the carbonyl of Tyr^298^. 

In the structure-function study performed by Kinney et al. in 2002 [32], an alanine scan of truncated goby U-II demonstrated that the replacement of Trp, Lys, and Tyr is crucial for the maintenance of biological activity. The sequence Trp-Lys-Tyr within U-II is essential for binding and activation of the receptor, indicating that the hydrophobic side chains of Trp^7^ and Tyr^9^ and the positive charge of Lys^8^ represent pharmacophoric elements. Moreover, NMR studies performed by Flohr et al. in 2002 [24] revealed that the distances between the pharmacophoric points are key elements in the development of SAR. Accordingly, first NMR studies applied to the receptor-unbound hU-II in water were developed in order to provide for a putative agonist pharmacophore. Structural model showed the Lys^8^ amino group and Tyr^9^ aryl ring being in proximity with a distance of 6.4 Å; the Trp^7^ and Tyr^9^ were separated by 12.2 Å, and Trp^7^ aromatic residue and Lys^8^ amino group were separated by 11.3 Å. Later, another agonist pharmacophore model was performed by studying conformation adopted by a less potent analogue without Val residue, that is, Ac-[Cys-Phe-DTrp-Lys-Tyr-Cys]-NH_2_ (200-fold less potent than hUT-II). Since this peptide was more closely similar to the receptor-bound conformation, this structural model suggested new distances between pharmacophoric elements of Trp-Lys-Tyr sequence (Lys^8^ amino group and Tyr^9^ aryl ring at 11.1 Å, Trp^7^ and Tyr^9^ aryl residues separated by 8.3 Å, and Trp^7^ aryl and Lys^8^ amino groups separated by 13.7 Å). Flohr et al. also studied the substitution of amino acids in the hexacyclic part of hU-II sequence with the corresponding D isomers. This led to dramatic decrease of the agonist activity, suggesting the importance of the side chains of these amino acids and their spatial orientation for interaction with the UT receptor; surprisingly, stereoinversion of L-Trp in D-Trp does not show a significant change of the EC_50_ value versus the endogenous ligand. The role of Lys^8^ was also investigated by replacing it with lipophilic amino acids and hydrophilic nonbasic amino acids that produce inactive peptides [33]. Thus, positive charge represented by primary aliphatic amine of Lys in position 8 is essential for the biological activity. However, reducing the distance of the primary aliphatic amine from the peptide backbone led to a progressive reduction of both potency and efficacy. [Orn^8^]U-II analogue showed a weak contraction of rat aorta strips corresponding to about 20% of the U-II maximal effect at micromolar concentrations. In position 9 the –OH group of Tyrosine was proved to be replaced with –OCH_3_, –NO_2_, –CH_3_, –F, –H, and –NH_2_ obtaining any improvement in potency or efficacy, except for 3-iodo-Tyr residue that produced a full UT receptor agonist, 6-fold more potent than the natural peptide. 

A further and useful attempt to alter potency and efficacy versus UT receptor was represented by the introduction of nonnatural amino acids into the sequence [32]. Replacement of the Tyr residue with the bulkier 2-Nal [(2-naphthyl)-L-alanine] in the goby U-II sequence showed similar potency in agonist activity (with a value of EC_50_ = 0.34 ± 0.1 nM than the value for the goby U-II, EC_50_ = 0.17 ± 0.05 nM) in the functional assay and a 6-fold improvement in affinity in the binding assay (*K*_*i*_ = 0.04 ± 0.02 nM), presumably due to enhanced hydrophobic interactions in the tyrosine-binding pocket. In contrast, the Bip residue, [(2-biphenyl)-L-alanine] did not show equal result, confirming that larger groups are not well accommodated. In this work, Kinney et al. also provided for a novel agonist pharmacophore model through the docking of goby UT-II into a rat UT receptor homology model. According to this modelling study, the Lys^8^ amino group of the ligand was essential for the interaction with Asp^130^ on transmembrane helix TM3 of UT receptor, with the binding cavity drawn by the extracellular loops that strengthened the accommodation of the ligand. The distances characterized for the pharmacophoric sequence were 11.2 Å between the Lys^8^ amino and Tyr^9^ aryl groups, 8.4 Å between the indole Trp^7^ and Lys^8^ amine, and 8.2 Å between the Trp^7^ and Tyr^9^ aryl groups.

## 3. Peptide Ligands

As mentioned above, the human U-II is involved in several pathophysiological pathways of disorders especially regarding cardiovascular system along with the observation that the interaction U-II/UT receptor regulates the contractility and growth properties of cardiac and peripheral vascular vessels led to identifying selective ligands. In particular, since the modulation of the U-II system offers a great potential for therapeutic strategies related to the treatment of cardiovascular diseases, the research of selective compounds is more intriguing. 

Based on the search for a definitive pathophysiological role for U-II and its receptor in the cardiovascular homeostasis and aetiology of relating disorders, the design of suitable tool compounds of peptide or nonpeptide nature could be of significant utility. New molecules may assist in determining this role by the development of selective UT receptor antagonists. 

Therefore, first attempts came out from observations on somatostatin system, due to sharing sequence peculiarities between hU-II and somatostatin. Indeed, some somatostatin analogues such as PRL-2903 Table 1, H-4Fpa-c[Cys-Pal-DTrp-Lys-Tle-Cys]-Nal-NH_2_, resulted in the ability to block the hU-II-induced rat aorta ring tone at micromolar concentrations, although it showed low species selectivity [34]. Another peptide somatostatin analogue, described by Coy et al. in 2000, showed moderate affinity for UT receptor. This peptide, named SB-710411, H-Cpa-c[DCys-Pal-DTrp-Lys-Val-Cys]-Cpa-NH_2_, was able to inhibit U-II-induced contraction in rat isolated thoracic aorta in a surmountable manner (*pK*_*b*_ = 6.28) [35]. Exposure to 10 *μ*M SB-710411 causes a significant shift in the agonist concentration-response curve with no suppression of the *E*_max⁡_, suggesting that it acts as a competitive antagonist. In contrast, SB-710411 did not alter the contractile efficacy of angiotensin-II, phenylephrine, or KCl, although it potentiated the contractile response to endothelin-1 in the isolated rat aorta. However, since little was known about the pharmacology of this ligand in other species, Behm et al. in 2004 [36] reported their observations on the pharmacological effects at the rat and monkey recombinant UT receptor because of the weak homology between rodent and primate UT receptors (~76% of homology than the homology between monkey and human UT receptor that are about 97% identical) [37]. SB-710411 acts as a ligand for both the recombinant rat and monkey UT receptor (30–150 nM affinities, 56- and 87-fold less potent than U-II, resp.). However, functional behaviour of this peptide at these two UT receptor orthologous differs radically. As was reported previously, SB-710411 itself did not promote inositol phosphate formation but inhibited the agonistic actions of U-II in the rat aorta [35]. In contrast to the rat, SB-710411 behaved as a full agonist at the recombinant monkey UT receptor, inducing the maximal response although EC_50_ was approximately 100-fold less potent than U-II. Thus, despite the antagonistic effects of SB-710411 observed at the rat UT receptor, this peptide acts as a full agonist at the monkey UT receptor. These findings suggest that the functional response of UT receptor modulators at the rodent UT receptor does not necessarily predict the functional response at nonrodent UT receptors, and the incoherence could result from alterations in receptor number and/or coupling efficiency as well as Camarda et al. in 2002 [38] proposed for a different UT receptor ligand, [Orn^8^]hU-II.

As cyclosomatostatin octapeptide analogue that shares structural similarities with SB-710411, the peptide neuromedin B receptor antagonist BIM-23127, H-D(2′)Nal-c[Cys-Tyr-DTrp-Orn-Val-Cys]-(2′)Nal-NH_2_ [39], was investigated by functional activity at recombinant and native UT receptors [40]. The effect of increasing concentration of BIM-23127 on hU-II-induced intracellular calcium mobilization in HEK293 cell lines expressing either the human or rat UT receptor was evaluated by generating hU-II concentration-response curves. These were indeed shifted progressively to the right in a parallel manner with no changes in the maximum response to hU-II, suggesting competitive antagonism. Moreover, BIM-23127 showed about 0.5 log unit lower affinity in competition binding experiments to human or rat UT receptors and inhibition of hU-II-promoted intracellular calcium mobilization producing a significant suppression of the maximum contractile response to hU-II. In contrast of this noncompetitive antagonism of contraction to U-II in isolated rat aorta, BIM-23127 inhibited calcium mobilization in human embryonic kidney 293 cells expressing UT receptors in a competitive manner. A related neuromedin B receptor antagonist, BIM-23042, H-DNal-c[Cys-Tyr-DTrp-Lys-Val-Cys]-(2′)Nal-NH_2_, displayed different functional activities at several UT receptor orthologues. It behaved as a full agonist at human and monkey UT receptor, a partial agonist at mouse UT receptor, and a competitive antagonist at rat UT receptor [41]. 

Among the most potent compounds, Camarda et al. in 2002 [38] identified the hU-II derivative [Orn^8^]U-II, that was characterized *in vitro* as a novel peptide ligand for the UT receptor. Modification of Lys^8^ to the nonnatural amino acid Orn was suggested by chemical observations that Lys belongs to the most important sequence for the biological activity. This synthetic analogue behaved as a full agonist in the calcium functional assay in HEK293 human and rat UT cells, inducing similar maximal effects as U-II. However, the potency of [Orn^8^]U-II at both receptors was 3-fold lower than U-II (*p*EC_50_ = 7.93 ± 0.16 and 8.06 ± 0.22, resp., whereas U-II increased intracellular calcium levels in HEK293 hUT and rUT cells with similar high potencies, *p*EC_50_ = 8.51 ± 0.18 and 8.54 ± 0.14, resp.). In contrast, different results were obtained in the rat aorta bioassay, in which the compound behaved as a competitive antagonist, showing only in highest concentrations (10 *μ*M) a weak residual agonist activity (25% compared to the maximal effect of U-II). The variance between results obtained between the cell and tissue assay could be interpreted assuming that [Orn^8^]hU-II is a partial agonist. 

In 2002 Grieco et al. [29] generated a novel peptide UT receptor agonist by introduction of an unusual amino acid in the disulphide bridge of hUT-II_(4–11)_, the potent analogue [Pen^5^]hU-II_(4–11)_, also known as P5U. This *β*,*β*-dimethyl-substituted cysteine residue led to higher conformational rigidity in the sequence of hU-II C-terminal octapeptide. Data showed that P5U has a 3-fold higher affinity for the UT receptor than the endogenous ligand as competition experiments witnessed its ability to displace the iodinated radioligand with comparable affinity (iodinated hU-II bound the human UT receptor saturably with high affinity, *pK*_*D*_ = 9.2 ± 0.14, whereas P5U has similar affinity to the unmodified C-terminal octapeptide hU-II_(4–11)_, *pK*_*i*_ = 9.7 ± 0.07). In functional experiments on the rat aorta, P5U was 20-fold more potent than hU-II and 10-fold more potent than hU-II_(4–11)_, being by far the most potent U-II analogue in the rat thoracic aorta bioassay. Interestingly, conformational analysis by [^1^H] nuclear magnetic resonance (NMR) spectroscopy combined with molecular modelling on this peptide also indicated further details about structure-activity relationships since the putative pharmacophoric Trp-Lys-Tyr sequence into the cyclic portion of this analogue, retained as the most important for full agonist activity, maintains the same spatial orientation as in the native peptide. Thus, the chemical modification brought by the unusual more constrained penicillamine residue mainly influences the proximal Phe^6^ position, leaving Trp, Lys, and Tyr residue nearly unaffected. The enhanced pharmacological properties observed in the case of P5U can be assigned to this conformational restriction revealing the importance of the exploration of specific orientations in the three-dimensional space by which amino acid side chains can interact with the receptor. 

Since antagonist peptides such as SB-710411, [Orn^8^]U-II, BIM-23127 so far described showed weak potency at UT receptors with concomitant antagonist activities to different receptor types and behaved as partial agonist activity at UT, new attempts were challenged in order to develop more potent and selective UT receptor antagonist. Patacchini et al. in 2003 [42] described the pharmacological activities of two compounds: [Pen^5^, Orn^8^]hU-II_(4–11)_ and [Pen^5^, DTrp^7^, Orn^8^]hU-II_(4–11)_, named urantide (urotensin-II antagonist peptide). Both peptides derived from the hU-II_(4–11)_ fragment, previously reported as the minimal active sequence of hU-II, as well as further replacement of Cys^5^ by penicillamine, *β*,*β*-dimethylcysteine, were achieved in order to give them conformational rigidity stabilizing the putative bioactive conformation. In functional experiments both peptides showed no agonist effect by cumulative administration in the range 0.1 nM to 10 *μ*M. However, urantide was totally ineffective as an agonist even when administered as a single concentration, that was not shown for [Pen^5^, Orn^8^]hU-II_(4–11)_, suggesting a sort of desensitization known to affect UT receptor-mediated responses in this preparation. As the most potent UT receptor antagonist compound so far reported in the rat isolated aorta, urantide has also high affinity for the human (*pK*_*i*_ = 8.3) and for the rat (*pK*_*i*_ = 8.3) UT receptors. Conformational studies on urantide performed in 2005 by Grieco et al. [43] showed that the distance between Trp^7^ and Tyr^9^ side chains was 11.5 Å, greater than that observed in peptide agonist P5U (6.1 Å) because of the inversion of L-Trp^7^ into the corresponding D isomer in urantide. The feature of inversion of the configuration of the Trp residue in position 7 was suggested by the presence of the same modification in both BIM-23127 and SB-710411. Urantide represented an extremely potent UT receptor antagonist since it was about 50- to 100-fold more potent than any other compounds tested in the rat isolated aorta. Despite of the potent UT receptor antagonist activity in the rat aorta bioassay, urantide showed residual agonist activity at human recombinant assay in a calcium mobilization assay [44]. In order to develop a selective antagonist, chemical modifications led to generating the peptide [Pen^5^, DTrp^7^, Dab^8^]U-II_(4–11)_, also known as UFP-803, closely related to the urantide sequence[45]. In the present molecule, the residual agonist activity is less than that of urantide. In the rat aorta bioassay, UFP-803 competitively antagonizes U-II contractile action behaving as a selective UT receptor antagonist. 

In 2003 a report from Sugo et al. [46] demonstrated the existence of a paralogue of U-II named U-II related peptide (URP), a novel peptide first isolated from the extract of rat brain and subsequently also proposed as endogenous ligand for UT receptor in the rat, mouse, and possibly in human. The amino acid sequence was determined as H-Ala-c[Cys-Phe-Trp-Lys-Tys-Cys]-Val-OH and it exhibits high binding affinity for human UT receptor in transfected cell lines and high contractile potency in the rat aortic ring assay, suggesting that some physiological effects could be not completely attributed to U-II. In order to evaluate the correct orientation of amino acid side chains belonging to the cyclic region of URP in the activity of the peptide, each amino acid has been replaced with the corresponding stereoisomer in a D-scan analysis [47]. D-isomer substitution within the cyclic portion in replacement of Phe^3^, Lys^5^, and Tyr^6^ reduced binding affinity and contractile activity, confirming the primary role of this portion in receptor recognition. In contrast, the [DTrp^4^]URP analogue retained important binding affinity, suggesting relative tolerance in the interaction with the receptor by stereoinversion occurring in that position. [DTrp^4^]URP showed also reduced efficacy appearing to behave as a partial agonist with moderate potency and a full antagonist with low potency, indicating that point substitution of the Trp residue in U-II and URP sequence could lead to the development of antagonists. Therefore, Chatenet et al. in 2012 [48] provided the replacement of the indole moiety in URP in order to obtain promising antagonists. In particular, the introduction of the more hydrophobic uncoded amino acid Bip led to the novel antagonist urocontrin, [Bip^4^]URP. The main feature of this peptide was the ability to reduce the efficacy of hU-II but not URP-induced vasoconstriction in a rat aorta assay. Despite the structural homology between U-II and URP and their concurrent expression in several human tissues, recent studies have reported different actions for these two peptides such as cell proliferation [49] and distinctive myocardial contractile activities [50]. Therefore, the identification of more selective ligands should be helpful for the rational design of more selective molecules in order to clarify the role of U-II and URP in the urotensinergic system. 

Several peptide UT receptor antagonists such as urantide, [Orn^8^]U-II, UFP-803, BIM-23042, and SB-710411 exhibit contradictory actions in selected assay systems since they have showed antagonist properties in rat isolated aorta and partial agonist action by mobilization of intracellular calcium in specific recombinant UT receptor HEK/CHO cell systems. Similar observations upon this residual agonist activity have already been made by Kenakin in 2002 [51] and Camarda et al. in 2002 [38] that have proposed an “assay-dependent” agonism/antagonism resulted from different UT receptor expression and/or signal transduction-coupling efficiency, for example, depending on the receptor density and the efficiency of receptor couplings. For this reason identification of a novel and selective antagonist was achieved by examining ligand-evoked UT receptor agonism under conditions of both low and high receptor density and efficient coupling and amplification. In 2006 Behm et al. [52] described GSK248451, H-Cin-c[DCys-Pal-DTrp-Orn-Val-Cys]-His-NH_2_ [53, 54], as a potent UT receptor antagonist in all native mammalian isolated tissues retaining an extremely low level of relative intrinsic activity in recombinant HEK cells (4-5 fold less than observed for urantide). Furthermore, since GSK248451 represents a selective UT receptor antagonist by blocking the systemic vasopressor actions of exogenous U-II it became a suitable tool compound for further investigations concerning the role of U-II in the aetiology of mammalian cardiometabolic diseases. 

## 4. Nonpeptide Ligands

The use of peptides as drugs in a therapeutic approach is often problematic because of their poor oral and tissue absorption, and their low stability due to the rapid proteolytic cleavage by enzymes. The pharmacokinetic limits of peptides can be generally overcome by developing nonpeptide molecules, inspired to the main sequence of the peptide and in particular mimicking the specific secondary structure responsible for the biological activity. Regarding to the design of nonpeptide ligands, the conformation and the size of the peptide backbone is often difficult to mimic by using as scaffold organic molecules and modifications onto hydrophobic, steric, and electronic properties could generate potential active compounds and optimize their affinity and selectivity. Nonpeptide agonists and antagonists at the human UT receptor could be important tool compounds in determining the role of U-II and its derivatives in the urotensin system, and they have been developed in several studies. Specifically, the design and synthesis of selective receptor antagonists should be helpful to clarify the role of human U-II as a multifunctional peptide in mammalian pathophysiological functions. Additionally, a pure nonpeptide antagonist stable on *in vivo* administration could be very helpful to provide an alternative pharmacological strategy in different disease models.

**Figure 2:**
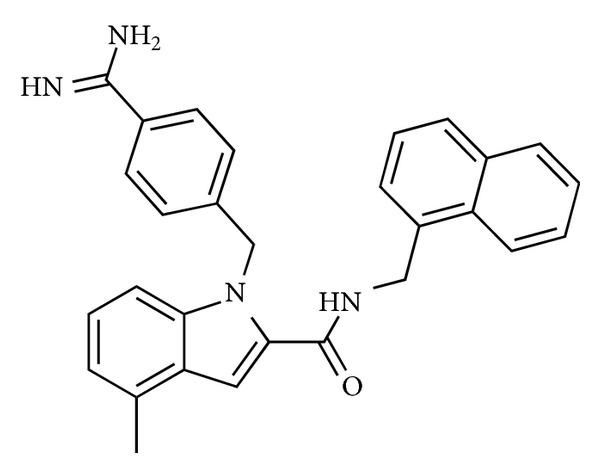
Structure of S7616.

Historically, first approaches for the discovery of new nonpeptide ligands at GPCR receptors were based on high-throughput screening (HTS) studies or knowledge of the 3D structure and secondary conformation adopted by the natural ligand. A virtual screening based on 3D pharmacophores defined from the key residues of U-II was even performed on an Aventis compounds database by Flohr et al. in 2002 [24] in order to identify functional antagonists of U-II. The screening was based on their two agonist pharmacophore models: one associated with the human U-II peptide and one associated with Ac-[Cys-Phe-DTrp-Lys-Tyr-Cys]-NH_2_. From 500 compounds that matched the U-II pharmacophore, the most notable compound was in S7616, 1-(3-carbamimidoyl-benzyl)-4-methyl-1H-indole-2-carboxylic acid (naphthalene-1-ylmethyl)amide, revealing an IC_50_ of 400 nM (Figure 2). 

The phenyl ring of the indole and the naphthalenemethylamine side chain are localized onto the two aromatic features of the pharmacophore. The basic benzamidine group in S6716 was shown to form a charged interaction with Asp^130^ residue within TM3 of the human UT receptor. Here, the basic amino group was considered as crucial feature for all following designed antagonists.

**Figure 3:**
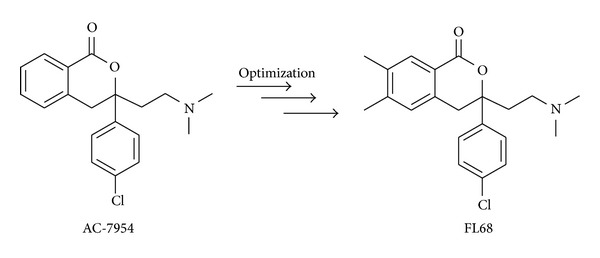
Structures of AC-7954 and its optimized derivative FL68.

Because of the absence of specific small molecule UT receptor agonists, Croston et al. performed a functional mammalian cell-based R-SAT assay for high-throughput screening in 2002 [55]. In this assay the UT receptor was multiplexed with vectors for the expression of additional receptor targets, such as the muscarinic M3 receptor and some orphan GPCRs, in order to increase the number of drug-target interactions tested without altering the response and sensitivity characteristics of potential ligands. Screening a library of 180000 small diverse organic molecules tested in a multiplexed R-SAT assay, AC-7954, 3-(4-chlorophenyl)-3-(2-(dimethylamino)ethyl)isochroman-1-one, was identified as a novel nonpeptide agonist with a potency of 300 nM at the human UT receptor (Figure 3). 

This compound is selective to activate the UT receptor, although the other receptors included in the multiplex screening assay belong to somatostatin and opioid receptor classes that are the closest to UT receptor for genetic sequence homology. Having no significant response when tested at concentrations up to 15 *μ*M on several receptor targets, including dopamine (D1, D2, D5), muscarinic (M1, M3, M5), serotonin (5-HT1A, 5-HT1B, 5-HT1D, 5-HT1E, 5-HT2A, 5-HT2B, 5-HT2C), histamine (H2), *β*-adrenergic (*β*-1, *β*-2), somatostatin (sst-2, sst-3, sst-5), CRF-1, CRF-2a, CRF-2b, *κ*-opioid, adrenomedullin, and CCK-a receptors, AC-7954 resulted to be a highly selective nonpeptide agonist of the UT receptor. This compound has low molecular weight, drug-like lipophilicity, a basic amino function (*pK*_*a*_ = 8.7), and it exhibits limited conformation flexibility for the presence of the bicyclic isochromane-based ring system. The product was synthesized as racemic mixture (*p*EC_50_ = 6.5) that was resolved in order to test enantiomers in R-SAT. Interestingly, the assay revealed that the isomer (+)-AC-7954 is more potent as UT receptor agonist (*p*EC_50_ = 6.6), indicating that this activity is highly stereoselective. The mode of interaction of (+)-AC-7954 with human UT receptor was clarified by later docking studies [31]. (+)-AC-7954 binds the human UT receptor through interaction between the basic amino group and Asp^130^ located on TM3 of the receptor as well as the p-chlorophenyl ring located in a hydrophobic pocket, where usually Tyr^9^ residue binds, and the benzo ring interacts with Phe^118^ by an aromatic stacking interaction. 

As first nonpeptide agonist the compound named AC-7954 has been considered as a lead compound and a series of analogues of this compound have been developed in order to obtain more potent nonpeptide ligands at the human UT receptor [56]. In accordance with this study the isochromanone core has been kept intact, whereas new bulkier amino groups and introduction of substituents in position 4 or in the aromatic rings were investigated so that the structure-activity relationship study around AC-7954 could complete the knowledge about U-II/UT receptor interaction. These several structural modifications led to both increased and decreased activities, although beneficial effects were obtained when substituents were introduced in the aromatic part of the isochromanone ring system, whereas more sterically demanding amino groups resulted to be damaging to the activity. The 6,7-dimethyl derivative of AC-7954 showed the most potency among the series (*p*EC_50_ = 6.87 ± 0.03) and once its racemate has been resolved into the pure enantiomers, it was indicated the (+)-enantiomer, subsequently named FL-68 (Figure 3), as the own potential active stereoisomer (*p*EC_50_ = 7.30). From the series of compounds synthesized in this study, FL-68 was found the most interesting since it was very active versus UT receptor and not showing at the same time any activity versus the closely related somatostatin receptors. 

**Figure 4:**
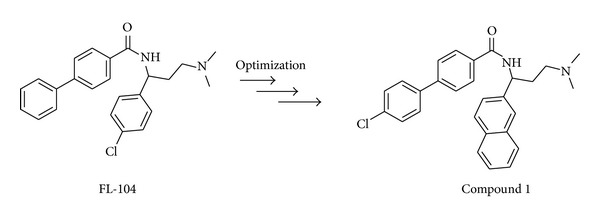
Structures of FL-104 and its optimized derivative compound 1.

Although the isochromanone-based agonists so far described were interesting for their druglikeness properties and their high selectivity for the UT receptor, in 2007 Lehmann et al. [57] chose to obtain new molecules by breaking of C3–C4 bond in the isochromanone scaffold. This synthetic strategy adopting for development of novel urotensin-II agonists was primarily focused to the introduction of different linkers between the two aromatic rings. Thus, a series of ether, ester, amide, sulfonamide, carbamate, and urea derivatives were performed. These more flexible compounds led to molecules most retained in activity and efficacy compared to AC-7954 except for ethers and sulfonamide probably due to the absence of conformational effects induced by the pharmacophoric carbonyl group. Interestingly, esters showed lower efficacy than other derivatives, whereas the introduction of larger and lipophilic substituents in the variable aromatic part of the molecule tend generally to increase potency. To investigate the improvement of the efficacy it seems that the introduction of electron-donating groups contributed positively. Furthermore, among benzamide series more lipophilic compounds were approximately 1 order of magnitude more potent than the other amides. Accordingly, the biphenylamide derivative, later known as FL-104, was recognized as a potent agonist (*p*EC_50_ = 7.49 ± 0.03) and its racemic mixture was resolved in order to evaluate the main active stereoisomer, identified in (+)-FL-104 isomer (*p*EC_50_ = 7.49), considerably more active than (−)-FL-104. The (+)-S-enantiomer of FL-104 was considered as one of the most potent nonpeptide agonists known (Figure 4). 

Analogues of FL-104 were designed and synthesized in order to enable comparisons between SAR in the isochromanone and benzamide series of UT agonists [58]. By the evaluation of the distance between the aromatic rings and the dimethylamino group and by the replacement of the dimethylamino group with a piperidine moiety new compounds were obtained and tested for their ability to stimulate the human UT receptor in an R-SAT assay. However, compound 1 was the most interesting among the series (Figure 4). Its racemate mixture was resolved and the (+)-(S)-enantiomer corresponded to the most active nonpeptide agonist compound with a *p*EC_50_ value of 7.64 ± 0.23, which is higher than the precursor FL-104.

**Figure 5:**
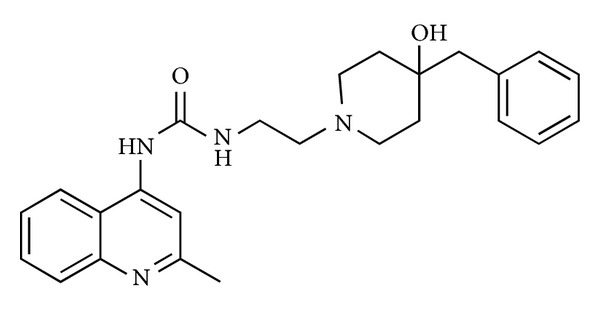
Structure of palosuran (ACT-058362).

As pharmacological tool in determining physiological and pathological roles of endogenous U-II in kidney diseases, in 2004 Clozel et al. [59] reported the discovery and characterization of a specific and potent inhibitor of the human UT receptor, the compound palosuran (ACT-058362) (Figure 5). 

Results from radioligand binding experiments carried out in membrane preparations from CHO cells expressing the human UT receptor indicated palosuran as potent inhibitor of ^125^I-U-II, binding at human UT receptor with an IC_50_ of 3.6 ± 0.2 nM, whereas radioligand ^125^I-U-II was potently inhibited by the unlabeled U-II with an IC_50_ value of 1.2 ± 0.2 nM. On cell lines such as TE-671 and CHO, the inhibitory binding potency of palosuran toward the human UT receptor was lower than on membranes (IC_50_ = 46.2 ± 13 nM on TE-671 cells and IC_50_ = 86 ± 30 nM on recombinant CHO cells). Binding studies showed also that the inhibitory binding potency of palosuran is higher more than 100-fold on human UT receptor compared with the rat UT receptor (IC_50_ = 3.6 nM toward the human receptor, IC_50_ value of 1475 nM for rat receptor). However, palosuran inhibited U-II-induced concentration of rat aortic rings in a potent and concentration-dependent manner and inhibited the maximal contractile response of the rings almost completely at 10^−4^ M suggesting an insurmountable kind of antagonism of contraction induced by U-II. Despite this, in binding studies using membrane preparations expressing human UT receptor, palosuran interacted competitively with its receptor. This controversial mode of inhibition observed between functional assays and receptor binding studies could be linked to the likely noncompetitive antagonism due to the partial internalization of the UT receptor after binding with palosuran. This could reduce the receptor availability on the cell surface in the isolated rat aortic ring system. Functional assays showed that palosuran was a selective antagonist of UT receptor not antagonizing the action of other vasoconstrictor agents such as KCl, endothelin-1, 5-hydroxytryptamine, and norepinephrine. Therefore, palosuran represents an important tool of nonpeptide nature in order to validate the role of endogenous U-II in disease models, in particular in kidney pathologies. For this reason, palosuran was considered as an interesting tool and a novel and interesting UT receptor antagonist for evaluating the pathophysiological role of endogenous U-II in renal system, since both U-II and UT receptor are highly expressed in the kidney [15]. Endogenous U-II plays a role in mediating the abnormal renal vasoconstriction after ischemia and short-term intravenous administration of palosuran reduced the glomerular and tubular dysfunction and renal tissue injury induced by renal ischemia [59].

**Figure 6:**
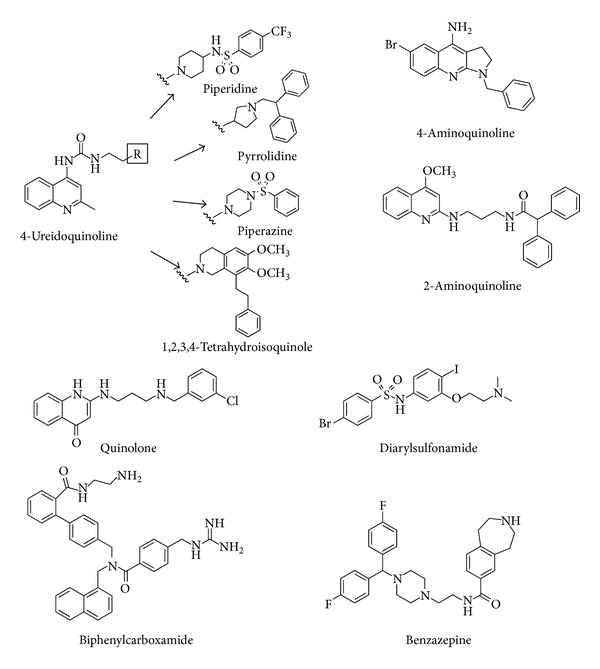
Structures of nonpeptide urotensin-II receptor antagonist reported in the patent literature.

Further evidence that the 4-ureido-quinoline core, also shared with the structure of palosuran, could be taken as a promising template for antagonists turned out from several patent applications. In addition, several examples of UT receptor antagonists have appeared in the patent literature but they were less discussed in this paper. Thus, 4-ureido-quinoline derivatives, in which 1,2,3,4-tetrahydroisoquinole, piperidine, piperazine, and pyrrolidine moieties were introduced, were also tested for their ability to displace human [^125^I]U-II binding to a rhabdomyosarcoma cell line (IC_50_ values ranging from 1 to 1000 nM) [60–62]. Other nonpeptide molecules reported in patent applications were based on 4-aminoquinolines [63] and quinolone, such as 2-aminoquinolines and 2-aminoalkylquinolin-4-ones derivatives [64], template (Figure 6). 

**Figure 7:**
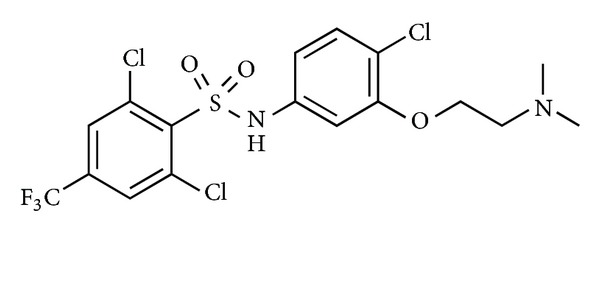
Structure of SB-611812.

Researchers at GlaxoSmithKline conducted extensive biological studies leading to the discovery of a series of arylsulfonamide derivatives developed from high-throughput screening. The compound SB-611812 showed potent binding at the rat UT receptor (*K*_*i*_ = 121 nM) and based on its antagonist activity in rat aortic tissue and interesting pharmacokinetic properties such as high bioavailability (~100%) and half-life (~5 h), this compound was proposed as a useful pharmacological tool (Figure 7). Accordingly, a coronary artery ligation study was conducted in rats with this lead UT antagonist since patients with congestive heart failure (CHF) are usually associated with high level of U-II and UT receptor in the heart tissue [65]. In this study, treatment with SB-611812 (30 mg/kg per day) for eight weeks significantly reduced overall mortality, left ventricular end-diastolic pressure, lung edema, right ventricular systolic pressure, central venous pressure, cardiomyocyte hypertrophy, and ventricular dilatation, underlying the importance of hUT-II in this disorder.

**Figure 8:**
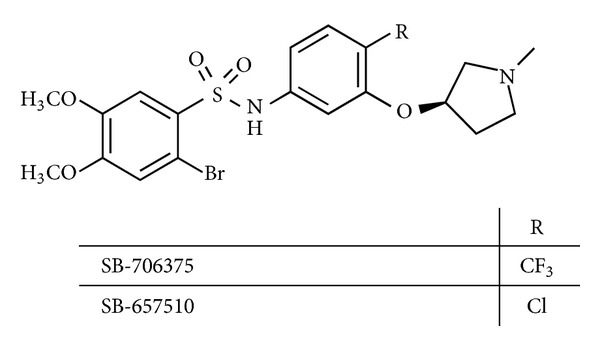
Structure of SB-706375 and SB-657510.

The identification and characterization of compound SB-706375 was originally described by Douglas et al. in 2005 [66] (Figure 8). This compound was identified as novel UT receptor antagonist, acting in a surmountable, reversible manner with high affinity across species (binding *K*_*i*_ = 4.7–20.7 nM) and good selectivity. The potent antagonist activity was demonstrated by its ability to inhibit [^125^I]hU-II binding to both mammalian recombinant and “native” UT receptors with a reversible mode of action (*K*_*i*_ = 4.7 ± 1.5 to 20.7 ± 3.6 nM at rodent, feline, and primate recombinant UT receptors; *K*_*i*_ = 5.4 ± 0.4 nM at the endogenous UT receptor in SJRH30 cells). The antagonist activity was also validated in a number of assays such as the inhibition of contraction of the rat isolated aorta (*pK*_*b*_ = 7.47) and the inhibition of intracellular calcium mobilization in HEK293 cells expressing UT receptor (*pK*_*b*_ = 7.29–8.00). SB-706375 was a selective U-II antagonist for the human UT receptor (≥100-fold) compared to 86 distinct receptors including ion channels, enzymes, transporters, and nuclear hormones (*K*_*i*_/IC_50_ > 1 *μ*M). The contractile responses induced in isolated aortae by KCl, phenylephrine, angiotensin-II, and endothelin-1 were unaltered by SB-706375 (at 1 *μ*M concentration). 

The very closely related compound SB-657510 [67] was also indicated as potent antagonist in isolated arteries from rats, cats, monkeys, and hUT-transgenic mice (Figure 8). The characterization of this compound led to the first nonpeptide UT receptor radiolabel, namely, the tritiated radiotracer [^3^H]SB-657510 [66].

Substituted diarylsulfonamides, reported in other patent applications, possessed significant affinity for UT receptors (*K*_*i*_ ~ 1 *μ*M). They were designed as UT receptor antagonists and CCR-9 antagonists for the treatment of congestive heart failure, stroke, ischemic heart disease, and so forth [68].

**Figure 9:**
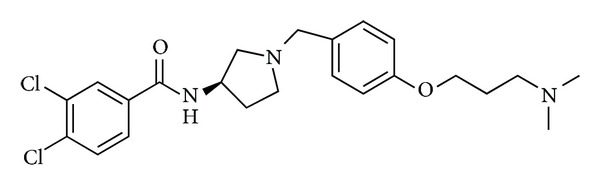
Structure of SB-436811.

Other UT antagonist series was represented by aminoalkoxy benzyl pyrrolidine derivatives even reported by GlaxoSmithKline. Based on an HTS protocol involving hU-II-mediated calcium mobilization in hUT-expressing HEK293 cells, the lead compound SB-436811 was identified for its moderate potency (*K*_*i*_ = 200 nM) binding to human UT receptor but weak potency in rat hUT binding (*K*_*i*_ = 3.2 *μ*M) [69] (Figure 9). As group linker between the substituted-phenyl moiety and the heterocyclic ring, the sulfonamide group was replaced with an alkyl group.

Biphenylcarboxamide and benzazepine scaffolds were also reported in patent applications since these derivatives demonstrated highly potent UT receptor antagonism [70]. In particular, most structurally different from other UT receptor antagonists, benzazepines represented one of the most potent antagonists at the human UT receptor so far described (IC_50_ ~ 2 nM). 

**Figure 10:**
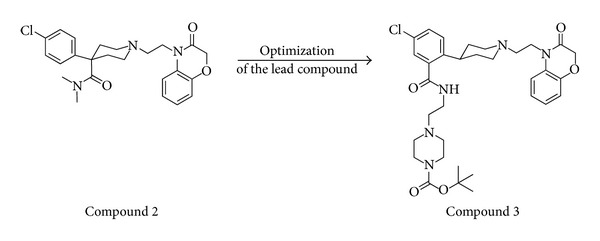
Structures of the lead compound 2 and its analogue compound 3 (piperidine derivatives).

With the aim to identify a compound with low nanomolar potency toward both rat and human UT receptor, as research tool for evaluating the role of U-II/UT receptor interaction in disease models, Luci et al. in 2007 [71] developed new series of small organic molecules. New lead structures were obtained by executing an HTS protocol involving a functional assay based on cells transfected with rat UT receptor, a fluorometric imaging plate reader (FLIPR) to measure intracellular calcium flux, and the potent peptide UT agonist Ac-c[Cys-Phe-Trp-Lys-(2′)Nal-Cys]-NH_2_ [32]. By the application of this assay to a large compound library, various 4-phenylpiperidine-benzoxazin-3-ones derivatives were identified. Compound 2, which contained a 4-(4-chlorophenyl)piperidine subunit, was identified as moderately potent compound (IC_50_ = 7.1 *μ*M). On the basis of this lead compound more analogues were elaborated and compound 3 (Figure 10) that, containing an aryl-substituted piperidine subunit, was found to be a more potent antagonist toward both rat and human UT receptor (IC_50_ = 10 nM at rat UT receptor and *K*_*i*_ = 65 nM at human UT receptor). For this reason, this compound was selected for *in vivo* evaluation and its efficacy was shown in reversing the ear-flush response induced by U-II in rats.

**Figure 11:**
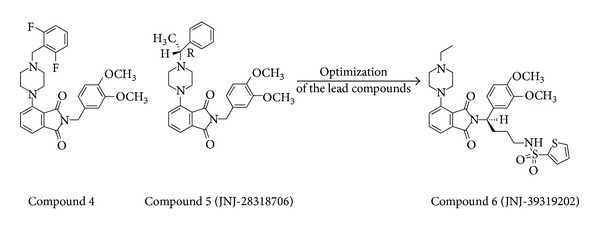
Structures of piperazino-isoindolinone derivatives compound 4 and compound 5 (JNJ-28318706) were optimized into the novel compound 6 (JNJ-39319202).

Other classes of compound that turned out from this screening were represented by piperazino-phtalimide and piperazino-isoindolinone derivatives, very different structural type of UT receptor antagonists from those previously reported in literature. Thus, Lawson and coworkers in 2009 [72] realized these novel series starting from the identification of the compound 4 (rat FLIPR EC_50_ = 0.54 nM, rat UT binding *K*_*i*_ = 0.12 nM) and the strictly related compound 5, also known as JNJ-28318706. The latter had improved metabolic stability and improved potency (rat FLIPR IC_50_ = 84 nM), and (R)-enantiomer exhibited also good oral bioavailability in pharmacokinetics experiments. However, as piperazino-isoindolinone derivative (Figure 11), the compound 6 (JNJ-39319202) showed single-digit nanomolar potencies in the rat FLIPR assay (IC_50_ = 1.0 nM) and in the human UT receptor binding assay (*K*_*i*_ = 4.0 nM). This compound also exhibited potent antagonism in the human calcium flux assay (IC_50_ = 8.0 nM). Moreover, a recent study reported a facile alkylation-cyclization reaction performed on isoindolinone core of JNJ-39319202 that yielded to novel tricyclic derivatives with retained potency in antagonism activity [73]. 

Aminomethylpiperazines derivatives were also drawn out from the structure belonging to *κ*-opioid receptor agonists [74]. In accordance with this study, optimization of the piperazine moiety provided high affinity urotensin-II receptor antagonists (more than 100-fold selectivity over the *κ*-opioid receptor) and, among this series, specific compounds inhibited urotensin-induced vasoconstriction in isolated rat aortic ring. 

Other 2-aminomethyl piperidine derivatives had moderate hUT binding affinity (*K*_*i*_ = 400 nM) and hUT functional activity (FLIPR IC_50_ = 600 nM) [75]. However, these series were correlated to problems such as cytochrome P450 inhibition and low oral bioavailability. Therefore, some improvements were obtained by using a piperazine core in such promising compounds. Subsequently, removal of the piperidine and piperazine linker groups led to a series of potent biphenylmethyl derivatives. 

**Figure 12:**
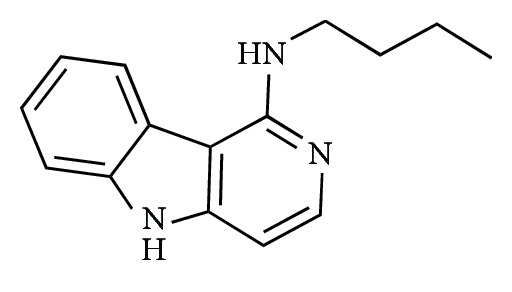
Structure of compound 7.

Wang et al. in 2008 [76] described a series of N-alkyl-5H-pyrido[4,3-*b*]-indol-1-amines as UT receptor antagonists since the tricyclic compound 7 was identified in an HTS as a lead compound due to its binding affinity (*pK*_*i*_ = 8.1) and submicromolar antagonist activity (*p*IC_50_ = 6.3) at human UT receptor (Figure 12). 

## 5. Conclusion

Despite of the large number of studies performed on U-II system, less knowledge even belongs to the issue regarding the pathophysiological role of urotensin-II/UT receptor interaction. Accordingly, several studies have reported contradictory activities resulting from the injection of exogenous U-II in the bloodstream. Vasoconstriction induced by exogenous U-II is indeed subjected to significant species and/or regional differences. Thus, from our point of view, the investigation of structure-activity relationships of U-II system represents the keystone that could address the unsolved question regarding the putative role of U-II in several disorders and physiological effects. The synthesis of U-II analogues has been performed adopting two main strategies based on the design and development of peptide or nonpeptide derivatives. Both the approaches present several advantages and disadvantages. Although peptides offer several advantages as therapeutic tools over small organic molecules, when they are composed of natural amino acids they are not very good drug candidates because of their intrinsic physicochemical and pharmacokinetic properties such as low bioavailability and biodistribution. On the other hand, as drug candidates in a therapeutic strategy, peptides offer greater efficacy, selectivity and specificity, and lower toxicity than small organic molecules since they represent the smallest functional part of a protein. However, the introduction of pharmacophoric elements in small organic structures is fascinating and favourable in the treatment of some diseases. Thereby, organic compounds represent the key for obtaining promising drug candidates by reaching easily a compromise between stability and selectivity. 

The research of the best therapeutic tools in disorders produced by U-II could be achieved by chemical optimization strategies for urotensin-II derivatives, peptides or nonpeptide analogues. The best knowledge of structure-activity relationships may provide useful chemical requirements in order to improve pharmacodynamic and pharmacokinetic properties such as bioavailability, reduction of elimination and biodegradation by proteolytic enzymes activity, low toxicity, reduced drug-drug interactions, and short half-life values avoiding accumulation of metabolites in tissues. As for urotensin peptides, substitution of natural amino acid residues by unnatural amino acid such as D-stereoisomer, nonproteogenic constrained amino acid or *β*-amino acid has represented strategic chemical approach resulting in the increase of stability and/or affinity for the UT receptor. At least, development of nonpeptide antagonists at the UT receptor is an attractive alternative as pharmacological tool in the identification of the role of endogenous U-II in several pathophysiological effects.

## Figures and Tables

**Table 1:** 

Name	Peptide sequence
PRL-2903	H-4Fpa-c[Cys-Pal-DTrp-Lys-Tle-Cys]-Nal-NH_2_
SB-710411	H-Cpa-c[DCys-Pal-DTrp-Lys-Val-Cys]-Cpa-NH_2_
BIM-23127	H-D(2′)Nal-c[Cys-Tyr-DTrp-Orn-Val-Cys]-(2′)Nal-NH_2_
BIM-23042	H-DNal-c[Cys-Tyr-DTrp-Lys-Val-Cys]-(2′)Nal-NH_2_
[Orn^8^]U-II	H-Glu-Thr-Pro-Asp-c[Cys-Phe-Trp-Orn-Tyr-Cys]-Val-OH
P5U	H-Asp-c[Pen-Phe-Trp-Orn-Tyr-Cys]-Val-OH
Urantide	H-Asp-c[Pen-Phe-DTrp-Orn-Tyr-Cys]-Val-OH
UFP-803	H-Asp-c[Pen-Phe-DTrp-Dab-Tyr-Cys]-Val-OH
URP	H-Ala-c[Cys-Phe-Trp-Lys-Tyr-Cys]-Val-OH
Urocontrin	H-Bip-c[Cys-Bip-Trp-Lys-Tyr-Cys]-Val-OH
GSK248451	H-Cin-c[DCys-Pal-DTrp-Orn-Val-Cys]-His-NH_2_
